# Synthesis of Calcium Peroxide Nanoparticles with Starch as a Stabilizer for the Degradation of Organic Dye in an Aqueous Solution

**DOI:** 10.3390/polym15051327

**Published:** 2023-03-06

**Authors:** Nurul Nazihah Amerhaider Nuar, Siti Nurul Ain Md. Jamil, Thomas Shean Yaw Choong, Intan Diana Mat Azmi, Nor Athirah Abdul Romli, Luqman Chuah Abdullah, Pen-Chi Chiang, Fan Li

**Affiliations:** 1 Department of Chemistry, Faculty of Science, Universiti Putra Malaysia (UPM), Serdang 43400, Selangor, Malaysia; 2 Centre of Foundation Studies for Agricultural Science, Universiti Putra Malaysia (UPM), Serdang 43400, Selangor, Malaysia; 3 Center of Sustainable Research, Department of Chemical and Environmental Engineering, Faculty of Engineering, Universiti Putra Malaysia (UPM), Serdang 43400, Selangor, Malaysia; 4 Institute of Tropical Forest and Forest Products (INTROP), Universiti Putra Malaysia (UPM), Serdang 43400, Selangor, Malaysia; 5 Graduate Institute of Environmental Engineering, National Taiwan University, Taipei City 10673, Taiwan

**Keywords:** calcium peroxide, nanoparticles, starch, stabilizer, Fenton reaction, wastewater

## Abstract

One of the most significant environmental problems in the world is the massive release of dye wastewater from the dyeing industry. Therefore, the treatment of dyes effluents has received significant attention from researchers in recent years. Calcium peroxide (CP) from the group of alkaline earth metal peroxides acts as an oxidizing agent for the degradation of organic dyes in water. It is known that the commercially available CP has a relatively large particle size, which makes the reaction rate for pollution degradation relatively slow. Therefore, in this study, starch, a non-toxic, biodegradable and biocompatible biopolymer, was used as a stabilizer for synthesizing calcium peroxide nanoparticles (Starch@CPnps). The Starch@CPnps were characterized by Fourier transform infrared spectroscopy (FTIR), X-ray diffraction (XRD), Brunauer–Emmet–Teller (BET), dynamic light scattering (DLS), thermogravimetric analysis (TGA), energy dispersive X-ray analysis (EDX) and scanning electron microscopy (SEM). The degradation of organic dyes, methylene blue (MB), using Starch@CPnps as a novel oxidant was studied using three different parameters: initial pH of the MB solution, calcium peroxide initial dosage and contact time. The degradation of the MB dye was carried out via a Fenton reaction, and the degradation efficiency of Starch@CPnps was successfully achieved up to 99%. This study shows that the potential application of starch as a stabilizer can reduce the size of the nanoparticles as it prevents the agglomeration of the nanoparticles during synthesis.

## 1. Introduction

**Figure 1:**
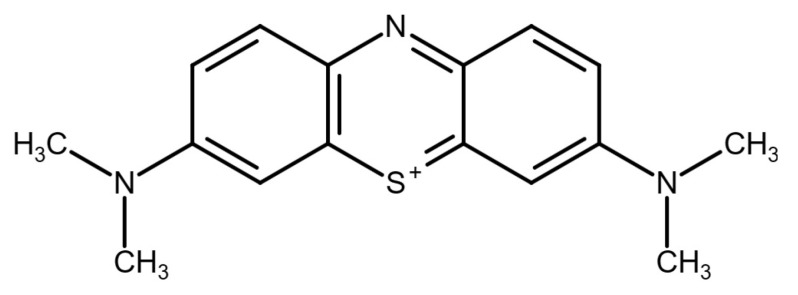
Chemical structure for methylene blue dye.

Dye residues found in wastewater can have detrimental effects on the environment and living organisms. Industries, such as cosmetics, printing, food processing, textiles and paper, are significant contributors to this issue. One particularly harmful dye is methylene blue (MB), a cationic dye commonly used in the dyeing industry (Figure 1). Due to its stability against heat and light, as well as its non-biodegradable properties, MB is difficult to remove from wastewater using traditional methods [1]. The discharge of MB dye and other colored effluents is a significant environmental problem, particularly in developing countries, as they can have toxic and carcinogenic effects on living organisms. Therefore, various methods have been developed for removing dyes from aqueous systems, such as adsorption or biosorption [2,3,4], electrocoagulation [5,6], ion exchange [7] and photocatalytic degradation [8,9] to mitigate this problem.

Among these methods, advanced oxidation processes (AOPs) are compelling ways to destroy a wide range of organic contaminants in wastewater. Various AOPs techniques have been applied for the degradation of MB, such as UV/H_2_O_2_ oxidation [10] and electrochemical oxidation [11,12]. Among these AOPs techniques, the Fenton reaction is regarded as an effective method to degrade organic dyes [13]. The decomposition of hydrogen peroxide (H_2_O_2_) in the presence of Fe(II) as a catalyst produced hydroxyl radical (•OH) which is categorized as a strong oxidative degradation of pollutants, as shown in Equation (1). However, the difficulty of transporting and the expensive cost of H_2_O_2_ have led researchers to look for other solid peroxide alternatives to produce H_2_O_2_.

Fe(II) + H2O2 → •OH + −OH + Fe(III)(1)

Calcium peroxide (CP) is a solid peroxide that exhibits stable H_2_O_2_ and O_2_ production when dissolved in water, as shown in Equations (2) and (3). CP shows significant degradation of various pollutants in aqueous systems, such as antibiotic compounds [14], sulfanamide [15], 4-nonylphenol [16] and 1,2-dichloroethane [17]. Previous studies by Madan et al. [18] have shown that the oxidation rate of organic molecules with calcium peroxide commercial (CP) is relatively slow. To solve this problem, CP must be synthesized in the form of nanoparticles to increase the surface area-to-volume ratio, thus enhancing the oxidation rate of pollutants. To achieve this, many scientists have used natural and synthetic polymeric materials as stabilizers and biotemplates to control the size of nanoparticles [19].

CaO_2_ + 2H_2_O → H_2_O_2_ + Ca(OH)_2_ (2)

(3) $${\mathrm{CaO}}_{2}+H_{2}O\;\to \;\frac{1}{2}O_{2}+\mathrm{Ca}{\left(\mathrm{OH}\right)}_{2}$$

Many polymers have been used as stabilizers in synthesis reactions of nanoparticles, such as polyethylene glycol (PEG) [20], chitosan [21,22], dextran [14,23], polyvinylpyrrolidone (PVP) [24], starch [25,26,27] and polyvinyl alcohol (PVA) [28]. The stabilizers will adsorb on the surface of the nanoparticles, preventing them from irreversible agglomeration over the precipitation process and forming stable dispersion by the increased steric repulsion [29]. Among them, starch has been used in several studies as a stabilizer of nanoparticles. Starch is a homopolymer of α-glucopyranose units with the chemical formula (C_6_H_10_O_5_)_n_ and composed of two polymer chains: amylose and amylopectin. The advantages of applying starch as a stabilizer are that it is relatively inexpensive, economical, environmentally friendly, benign and renewable. Starch can be found in various sources, such as cassava, tapioca, potato, maize, wheat and rice.

Starch can act as a coating/capping agent, functionalizing agent, stabilizing agent, pore-forming agent and coordinating agent in the synthesis of peroxide-based nanoscale materials [30]. Furthermore, the complex polymeric structure of starch, with its carbon-based spiral matrix and numerous polyol groups, creates a protective and functionalized environment that facilitates the formation of calcium ions in structures. The hydroxyl groups of amylose and amylopectin can participate in intramolecular and intermolecular supramolecular interactions and coordinate with the Ca^2+^ ions to keep the nanoparticles highly aggregated [24].

This study aimed to explore the feasibility of using starch as a stabilizer in the synthesis of calcium peroxide nanoparticles (CPnps). A modified chemical precipitation method synthesized the final product labeled as Starch@CPnps. The impact of starch on the Starch@CPnps was analyzed through various characterization techniques such as Fourier transform infrared spectroscopy (FTIR), X-ray diffraction (XRD), Brunauer–Emmett–Teller (BET), dynamic light scattering (DLS), energy dispersive X-ray analysis (EDX), and scanning electron microscopy (SEM). Finally, the degradation efficiency of Starch@CPnps as a novel oxidant was also assessed by measuring its ability to degrade MB dye under different conditions, including the initial pH of the MB solution, the initial dosage of CaO_2_ and contact time.

## 2. Materials and Methods

### 2.1. Materials

Calcium oxide (CaO, ≥99.9%), hydrogen peroxide (H_2_O_2_, 30%), starch ((C_6_H_10_O_5_)_n_), sodium hydroxide (NaOH), ethyl alcohol (C_2_H_5_OH, 95%), hydrochloric acid (HCl, 37%), methylene blue powder (C_16_H_18_ClN_3_S), iron (II) sulfate (FeSO_4_), potassium permanganate (KMnO_4_, 0.2M) and sodium sulphite anhydrous (Na_2_SO_3_, ≥98%) were purchased from R&M Chemicals (Semenyih, Malaysia). Manganese (II) sulfate (MnSO_4_, ≥99%) was purchased from Bendosen Laboratory Chemicals (Bendosen, Norway). Calcium peroxide (CaO_2_, 65%) was purchased from Alfa Aesar (Ward Hill, MA, USA). Solutions were prepared with distilled water. A digital pH meter (Sartorius PB-10, Sartorius, Goettingen, Germany) was used to measure pH. All chemicals were analytical grade.

### 2.2. Synthesis of Calcium Peroxide Nanoparticles with Starch as a Stabilizer (Starch@CPnps)

**Figure 2:**
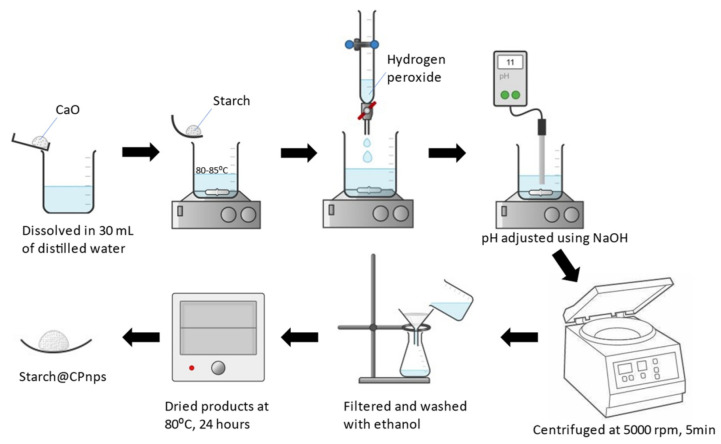
The schematic diagram for the synthesis of Starch@CPnps adapted from Amerhaider Nuar et al. [14].

The synthesis of Starch@CPnps was conducted using a modified chemical precipitation method, as described in our previously published article [14]. As shown in Figure 2, CaO was dissolved in 30 mL of distilled water, and the solution was sonicated to completely dissolve all the CaO powder. Then, the solution was heated up to 80–85 °C with a constant stirring at 300 rpm. Next, a certain amount of starch was added to the heated solution according to the weight ratio of starch and calcium oxide powder (1.0: 1.0). Then, 15 mL of 30% H_2_O_2_ was added dropwise into the mixture, and the solution was stirred for 2 h. In order to precipitate out the Starch@CPnps, 0.2M NaOH solution was added dropwise into the solution to form a basic medium (pH 10–11), and a beige precipitate formed. The precipitate was separated using centrifugation at 5000 rpm for 5 min and rinsed multiple times with ethanol. The final product was dried at 80 °C in an evacuated oven for 24 h.

### 2.3. Determination of Calcium Peroxide Content in the Starch@CPnps

**Figure 3:**
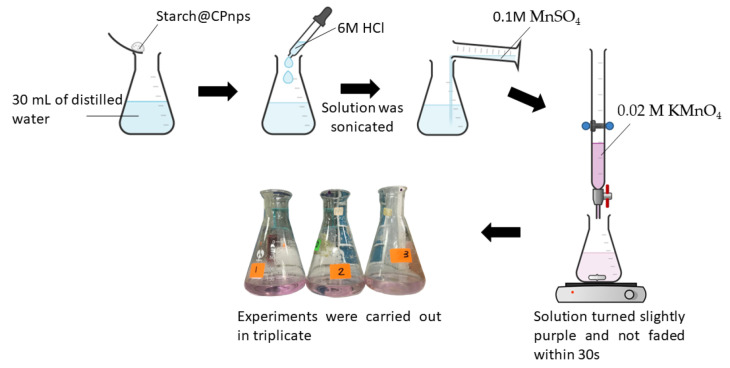
Schematic diagram on permanganate titration for determination of calcium peroxide content in Starch@CPnps.

Briefly, the Starch@CPnps sample was placed in an Erlenmeyer flask and dissolved in 30 mL of distilled water. Then, 10mL of 6M HCl was added into the solution, and the solution was sonicated to dissolve the entire sample. Next, 1 mL of 0.1M MnSO_4_ was added to the solution and titrated with 0.02M KMnO_4_ standard solution until the solution turned slightly purple and the color did not disappear within 30 s [31]. Figure 3 shows a schematic diagram of the permanganate titration for the determination of calcium peroxide content.

Calcium peroxide content in the synthesized Starch@CPnps was calculated using Equation (4):

(4) $$\mathrm{Calcium}\;\mathrm{peroxide}\;\mathrm{content}\;\left(\%\right)=\left(\frac{5}{2}\right)\times \left(\frac{\left[{\mathrm{KMnO}}_{4}\right]\times {\mathrm{volume}\;\mathrm{KMnO}}_{4}\;\mathrm{used}\times 72.08}{\mathrm{mass}\;\mathrm{of}\;\mathrm{CP}\times 1000}\right)\times 100$$

where [KmnO_4_] is the initial concentration of KMnO_4_, and 72.08 is the molar mass of CP. Experiments were run in triplicate. Equation (5) represents the permanganate reaction between hydrogen peroxide and permanganate ions under acidic conditions. From the equation, it can be seen that the reduction of the permanganate ion to Mn^2+^ by oxidation with hydrogen peroxide leads to the emission of oxygen gas and the formation of water. The reaction is assisted by manganese sulphate (MnSO_4_), which provides an excess of Mn^2+^ to prevent the oxidation of the chloride ions.

2MnO_4_^−^ + 5H_2_O_2_ + 6H^+^ → 2Mn^2+^ + 5O_2_↑+ 8H_2_O (5)

### 2.4. Degradation Experiments for CP Commercial and Starch@CPnps

Different parameters, including the initial pH of MB dye solution (3, 7 and 11 ± 0.1), initial dosage of the sample (0.05, 0.2, 0.4 and 0.6 ± 0.1 g), and contact time (0–60 min), were investigated in the degradation studies of MB dye. The experiments were carried out in a batch procedure using a series of Erlenmeyer flasks at room temperature (25 ± 2 ˚C). The MB dye was diluted from the stock solution to a concentration of 20 ± 0.1 ppm, and the pH of the solution was adjusted by adding either HCl (0.1M) or NaOH (0.1M) [32,33]. A specific amount of sample was added to the 100 mL of the MB dye solution, followed by the addition of 0.1 ± 0.01 g Fe(II). The solutions were then placed in a water bath shaker and shaken continuously at 150 rpm. At fixed time intervals, samples were taken out and centrifuged. The supernatant was then analyzed with a UV-Vis spectrophotometer (Halo DB-20, Dynamica Scientific Ltd., Livingston, UK) at 664 nm, the wavelength at which the MB dye shows maximum absorption. Using the standard curve provided by the calibration of the MB dye (R^2^ = 0.99), the absorbance was converted to concentration, and the percentage degradation of the MB dye was calculated using Equation (6):

(6) $$\mathrm{MB}\;\mathrm{dye}\;\mathrm{degradation}\;\mathrm{efficiency}\;\left(\%\right)=\frac{{\left[\mathrm{MB}\right]}_{o}-{\left[\mathrm{MB}\right]}_{t}}{{\left[\mathrm{MB}\right]}_{o}}\times 100$$

where [MB]_0_ and [MB]_t_ represent the initial concentration (20 ppm) and concentration of MB dye (ppm) at various time intervals (min), respectively.

### 2.5. Characterization of Starch@CPnps

The FTIR analysis of the synthesized Starch@CPnps was performed using FTIR instruments (Nicolet) in the range of 4000 to 500 cm^–1^. The phase analysis of the sample was evaluated using X-ray diffractometry (XRD) (Shimadzu XRD-6000, Kyoto, Japan) in the range of 2θ = 20° to 80°, using CuKα radiation and a step size of 4°/min. X’Pert HighScore Plus software version 2.2.4 (PANalytical B.V., Almelo, Netherlands) was used to analyze data from XRD, and the full width at half maximum (FWHM) of the peaks was calculated. The thermal stability of the sample was examined using Mettler Toledo TGA/SDTA 851 (Greifensee, Switzerland) in a nitrogen atmosphere, with a heating rate of 10 °C min^−1^ and at the temperature range of 50 °C to 600 °C. The size and polydispersity index (PDI) of the Starch@CPnps dispersed in ethanol were determined using a particle sizer (Nano S, Malvern Instruments Ltd., Malvern, UK) by measuring at a scattering angle of 90°. The surface morphology and elemental analysis were characterized using scanning electron microscopy (SEM) (NOVA NANOSEM 230, Hillsboro, OR, USA) magnification: 500–300,000). Additionally, Brunauer–Emmet–Teller (BET) analysis was conducted using Micromeritics (Quantachrome Co., Hampshire, UK) degassed at 80 °C for 24 h in a nitrogen environment (77 K).

## 3. Results and Discussions

### 3.1. Characterization of Starch@CPnps

**Figure 4:**
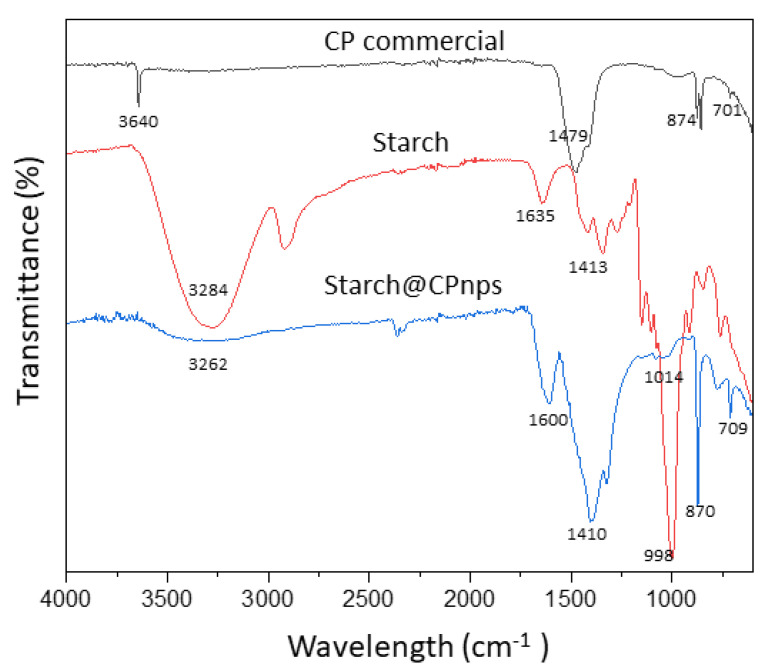
FTIR spectra of CP commercial, starch and Starch@CPnps.

Figure 4 shows the FTIR spectra of CP commercial, starch and Starch@CPnps synthesized by the modified co-precipitation method. For CP commercial, the distinct peak at 3640 cm^−1^ was attributed to the hydroxyl group’s vibrational mode of the O—H bond, which may originate from water absorbed from the humid environment. An intense peak can be seen in the spectra located between 1479 cm^−1^. This peak denoted the presence of the carbonate group of calcites [34]. Moreover, from the CP commercial spectra, two small common absorption peaks at 701 cm^−1^ and 874 cm^−1^ can be seen, respectively, appeared due to O—O stretching vibrations of peroxide. As for starch spectra, a broad peak appeared at 3284 cm^−1^ which was attributed to the stretching vibrations of O—H groups potentially including hydroxyl groups from water, alcohol, phenol and carboxylic acid.

Moreover, a small peak at 1635 cm^−1^ represents the hydroxyl group of chemisorbed and/or physisorbed H_2_O. Peaks at 1413 cm^−1^ are attributed to the C—H bending and angular deformation of the C—H bond in the starch molecules [35]. An intense peak at 998 cm^−1^ can be assigned to the skeleton vibrational mode of glycosidic linkage C—O and C—H stretching vibrations of starch molecules [36]. Furthermore, the impact of incorporating starch in the synthesis of Starch@CPnps has been shown in the FTIR spectra. The presence of starch in Starch@CPnps was confirmed by the absorption peaks at 3262 cm^−1^, 1600 cm^−1^, 1410 cm^−1^ and 1014 cm^−1^. Furthermore, a prominent peak can be observed at 1410 cm^−1^, attributed to the carbonate group (CO_3_^2−^) in calcites. The peak at 1600 cm^−1^ appeared due to the presence of hydroxyl group from moisture in the sample. In addition, two absorption peaks at 709 cm^−1^ and 870 cm^−1^ appeared to correspond to the O—O stretching vibration of peroxide [37].

**Figure 5:**
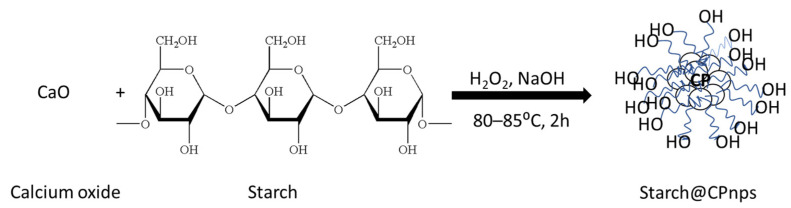
Proposed adsorption mechanism of starch onto CPnps via hydrogen bonding.

The results of FTIR analysis of Starch@CPnps suggest that the interaction between the hydroxyl groups of starch and the surface of the CPnps is likely due to hydrogen bonding, as illustrated in Figure 5. The formation of both inter- and intramolecular hydrogen bonds between starch and the surface of CPnps is crucial and is facilitated by the presence of water molecules. This is evident in the O—H bands’ merging and broadening at 3262 cm^−1^ from Starch@CPnps spectra.

**Figure 6:**
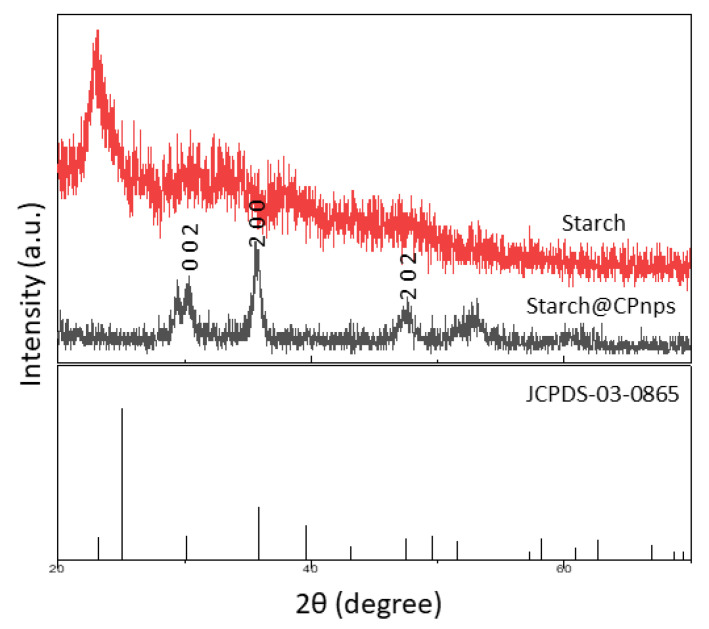
X-ray diffraction patterns of starch, Starch@CPnps and the standard pattern for CaO_2_.

The XRD patterns of the synthesized Starch@CPnps and CP commercial are shown in Figure 6. The XRD patterns for Starch@CPnps show dominant peaks at 2θ = 30.28°, 35.80° and 47.57°. These peaks are in agreement with the standard pattern for CaO_2_ by the Joint Committee on Powder Diffraction Standards (JCPDS-03-0865) and are consistent with the results of previous studies [38,39]. As the prominent diffraction peak in the (2 0 0) plane by Starch@CPnps, better crystallinity in Starch@CPnps than starch has been observed. Hence, adding starch as a stabilizer has confirmed its supportive nature towards Starch@CPnps formation. Furthermore, extra diffraction peaks corresponding to the peak of calcite at 2θ = 29.37° and 51.25° were also detected in the XRD pattern of Starch@CPnps [20]. The presence of calcites, identified as an impurity in the synthesis of Starch@CPnps, was confirmed through FTIR analysis. Calcite production likely occurred due to the carbonation of calcium hydroxide formed during the hydrolysis of precipitated Starch@CPnps in the synthesis process.

The average particles sizes of Starch@CPnps were calculated using the Debye–Scherer equation, as shown in Equation (7):

D = Kλ/(βcosθ)(7)

where K is the Debye–Scherrer constant (0.9), D is the average size for the nanoparticles (nm), λ is the wavelength of the CuKα radiation, θ is the angle of Bragg in radians and *β* is the FWHM of the XRD peak at the diffraction angle. The average size of Starch@CPnps was determined to be 4.76 nm, calculated using the intense peak at 2θ = 35.8° with a d-spacing of 2.52 Å. The small value of the average sizes for the Starch@CPnps sample could be due to the addition of starch, which prevents the agglomeration of the nanoparticles in the solution.

**Figure 7:**
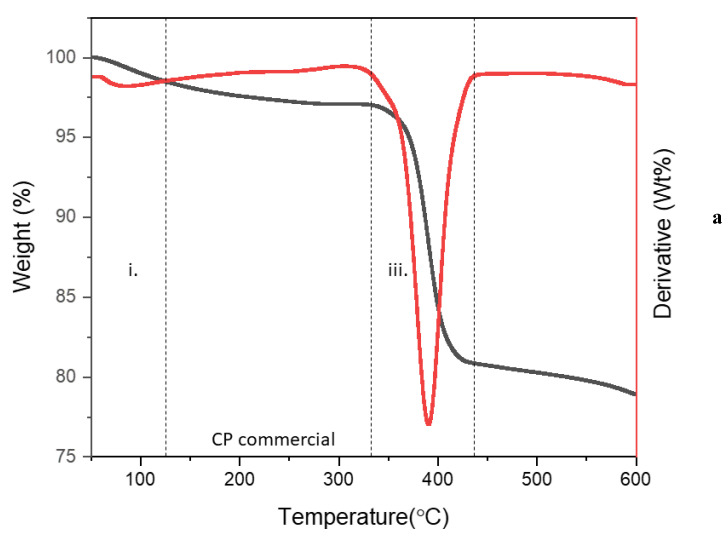

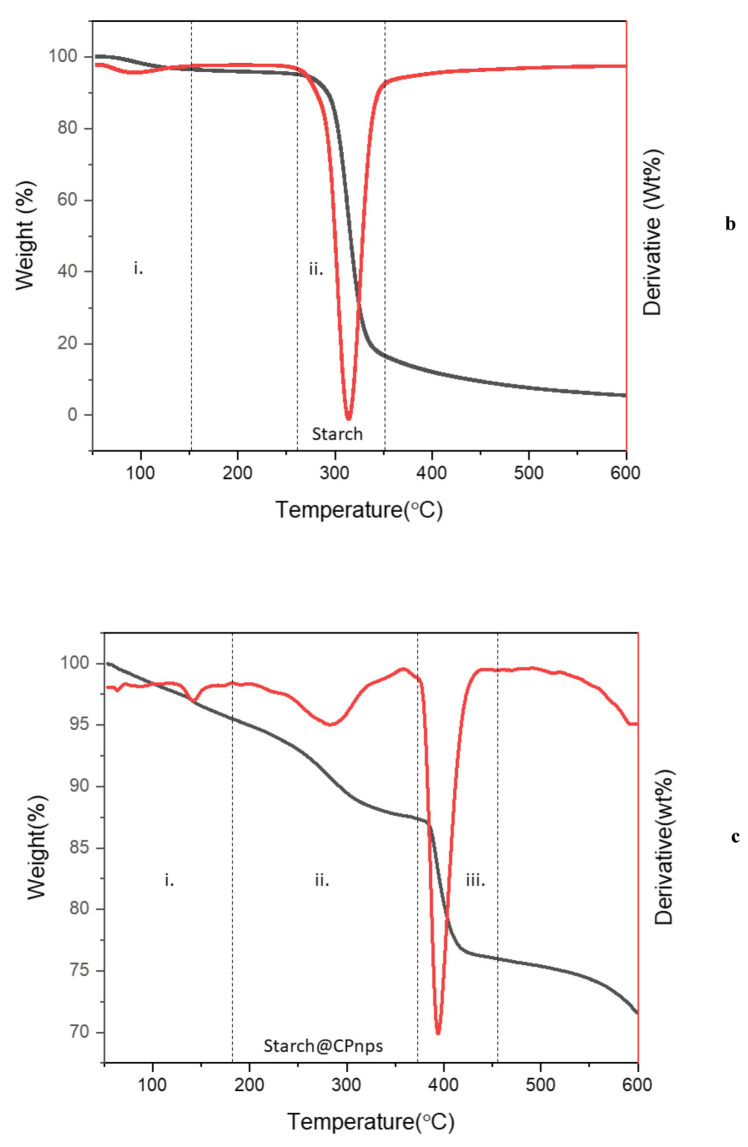
TGA and DTG curves of (**a**) CP commercial; (**b**) starch; and (**c**) Starch@CPnps. All the labelled stages in the figures are explained in Table 1.

TGA and its first derivatives (DTG) were utilized to determine the thermal stability of CP commercial, starch and Starch@CPnps, as shown in Figure 7. The thermal decomposition of the studied samples can be divided into three stages, as tabulated in Table 1, consisting of (i: 50–225 °C, ii:183–357 °C and iii: 353–454 °C). As demonstrated in Figure 7a, the first stage, (i), of the thermal degradation of CP commercial at 88°C resulted in a 3.1% weight loss due to the evaporation of water molecules. In the third stage, (iii), a weight loss of 17.3% was observed due to the decomposition of CaO_2_ at 390 °C [40]. The thermal degradation of starch (Figure 7b) was observed to occur in two significant stages. The first stage, (i), which occurred at 100 °C, was primarily due to the dehydration of water and removal of impurities, resulting in a weight loss of 4.4%. The second stage, (ii), between 256 °C and 351 °C, involved the decomposition of the polysaccharide chains in the starch, resulting in a weight loss of 85%. This breakdown was caused by various processes, including the rupture of polysaccharide chains through dehydration, deamination, deacetylation, breaking of glycoside bonds and opening of pyranose rings [41]. The thermal degradation process of Starch@CPnps occurs in three distinctive stages, as shown in Figure 7c. The initial phase, (i), is characterized by the dehydration of water molecules present within the sample and the breakdown of any impurities with a weight loss of 4.5%. The second stage of degradation, (ii), which occurs within a temperature range of 183–357 °C, corresponds to the degradation of the polysaccharide chain of starch, resulting in a weight loss of approximately 9.3%. Finally, the third phase, (iii), at a temperature of 394 °C is attributed to the decomposition of calcium peroxide, resulting in a weight loss of around 11.6%.

Additionally, the amount of CaO_2_ in the synthesized Starch@CPnps was determined by using the stoichiometry of the decomposition reaction given in Equation (8) and the formula outlined in Equation (9) [20].

2CaO_2_ → 2CaO + O_2_ (8)

(9) $$\mathrm{CP}\;\left(\%\right)=\frac{\Delta m}{m_{o}}\times \left(\frac{72.08}{0.5\times 32}\right)\times 100$$

where ∆m is the difference between the initial and final mass of sample (g), and m_o_ is the initial mass of the sample (g). The ratio of 72.08:32 is the molar mass of CP to the eliminated oxygen, and the number of moles of released oxygen for each mole of the sample was assumed to be 0.5. The CP content for the Starch@CPnps sample calculated by the above method was 67.6%, coinciding with the purity determination by permanganate titration (Table 3).

**Figure 8:**
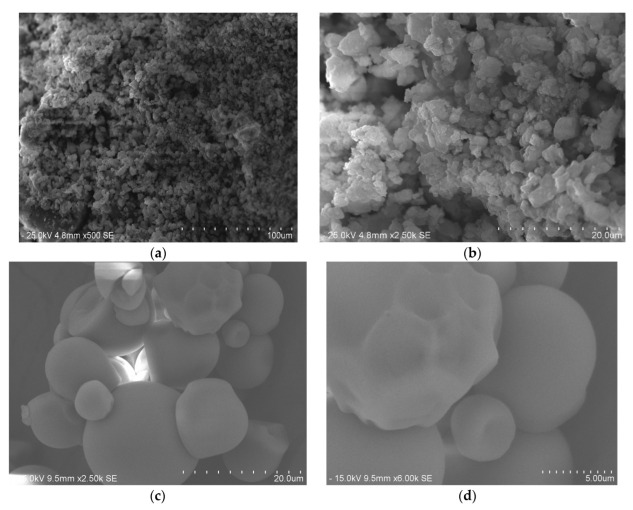
SEM images of (**a,b**) Starch@CPnps and (**c,d**) starch at different magnifications.

**Figure 9:**
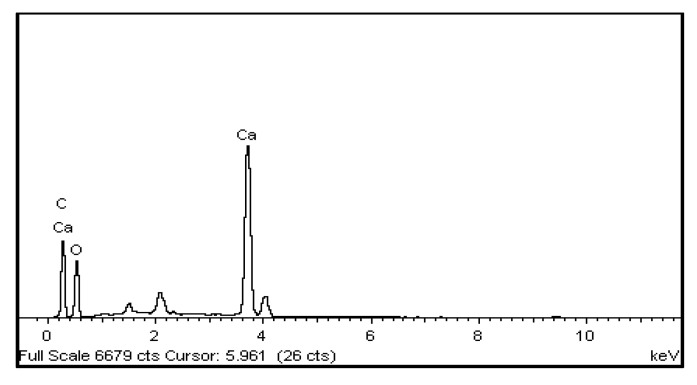
Energy-dispersive X-ray spectra recorded for Starch@CPnps.

Figure 8a,b depict the morphology and structure of Starch@CPnps and Figure 8c,d for starch at various magnifications. The irregular shapes of Starch@CPnps, as seen in the SEM images, were likely caused by the disruption of their spherical morphology during sample collection from the crucible with a spatula, contrary to the spherical shape observed in the starch images. Table 2 shows elemental data for Starch@CPnps, while Figure 9 shows EDX images for Starch@CPnps. The lower atomic percentage of calcium in Starch@CPnps compared to carbon and oxygen is probably due to the different atomic masses of these elements. In addition, the presence of starch in the sample may affect the atomic percentage of each element. Starch is a complex carbohydrate composed primarily of glucose units with carbon, hydrogen and oxygen atoms. The presence of starch in the sample will increase the relative proportion of carbon and oxygen, which decreases the relative proportion of calcium in the sample.

**Figure 10:**
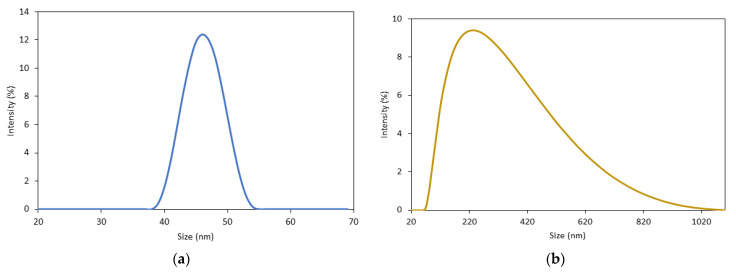
Particle size distributions of (**a**) Starch@CPnps and (**b**) CP commercial.

The particle size and distribution of Starch@CPnps and CP commercial were analyzed using dynamic light scattering (DLS). As shown in Figure 10a,b and Table 2, the analysis reveals that Starch@CPnps has a narrow particle size distribution, whereas the commercially available CP has a broad particle size distribution. The polydispersity index (PDI) values for Starch@CPnps and CP were 0.325 and 0.561, respectively. A PDI value less than 0.5 suggests that the nanoparticles are monodisperse and aggregatively stable. The use of starch as a stabilizer during the synthesis of Starch@CPnps contributed to the lower PDI value and the alignment of primary nanoparticles to form aggregates of uniform and controllable size. This creates a steric effect that slows down the agglomeration process and lowers the surface energy of Starch@CPnps.

Table 3 shows the physicochemical properties of CP commercial and Starch@CPnps, which consist of CaO_2_ content from permanganate titration, BET and DLS analysis. The BET analysis was used to compare the specific surface area, pore size and pore volume of Starch@CPnps with CP commercial. For Starch@CPnps, it shows that the average content of calcium peroxide in the product obtained was 72.3%. The CaO_2_ content of the synthesized Starch@CPnps is consistent with CP commercial, as the CaO_2_ content of these products, such as 466271 Sigma Aldrich and 21157 Alfa Aesar, are approximately 65–75%. Furthermore, the results show that Starch@CPnps have a larger specific surface area compared to CP commercial. Specific surface area is a measure of the total surface area of nanoparticles per unit mass. A larger specific surface area corresponds to a larger pore size and pore volume for Starch@CPnps compared to CP commercial. The small particle size of Starch @CPnps contributes to the larger surface area, while the varying size of CP particles results in a smaller surface area and a bigger average size. The surface area of a material is a measure of the total area of the surfaces of its particles. As Starch@CPnps has a larger surface area compared to CP commercial, this can be attributed to the application of starch as a stabilizer, which prevents agglomeration of the nanoparticles during the synthesis step. The larger surface area of Starch@CPnps is beneficial to improve the degradation of MB dye performance in a later section.

### 3.2. Methylene Blue Degradation Analysis

(i) Effect of Initial pH

**Figure 11:**
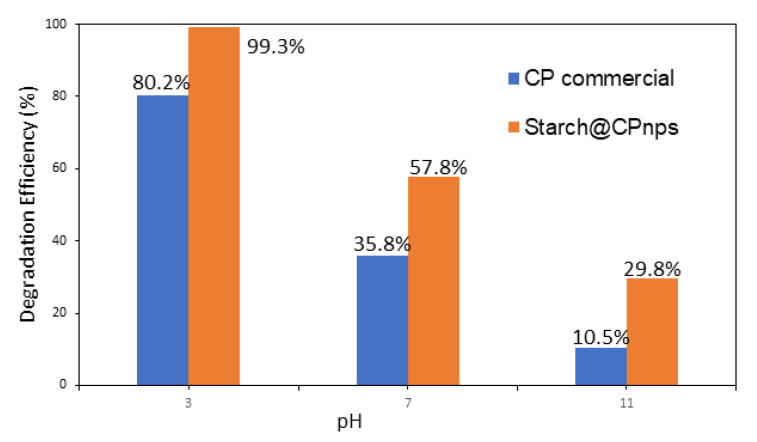
Effect of pH on the degradation efficiency of the MB dye; initial dosage of CaO_2_ = 0.6 g, [MB]_o_ = 20 ppm, Fe(II) dosage = 0.1 g and contact time of 60 min.

**Figure 12:**
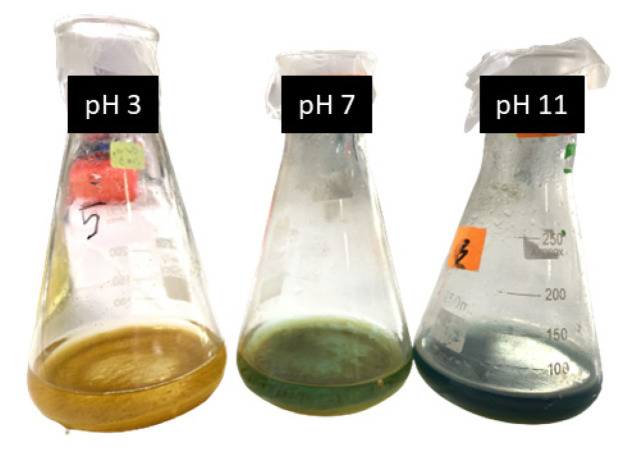
The effect of different pH (3, 7 and 11) on decolorization of MB dyes.

Figure 11 shows the impact of pH on the degradation rate of MB dye using the Fenton reaction. It was found that acidic conditions are the most influential pH for the generation of hydroxyl radicals (•OH). A range of pH levels between 3 and 11 was tested by adjusting the solution with HCl or NaOH, and the results are shown in Figure 10. The degradation results indicate that the optimal pH for maximum degradation of MB is pH 3. The Fenton reaction was performed for 60 min using CP commercial and Starch@CPnps samples, with constant sample dosage (0.6 g), a dosage of Fe(II) (0.1 g) and at room temperature (25 ± 2 °C) with 20 ppm of methylene blue dye. It was observed that Starch@CPnps performed better than CP commercial at all pH values, with the best performance at pH 3. According to the literature, the Fenton reaction, which is used to degrade the methylene blue (MB) dye, is a pH-sensitive process [42]. Thus pH value of the solution can significantly impact the reaction’s speed for pollution degradation. Specifically, the reaction is most efficient at acidic values between pH 3 and 4 as acidic conditions can effectively generate hydroxyl radicals (•OH), which are necessary for the oxidation of the MB dye. When the pH of the solution is too high, Fe(II) used in the Fenton reaction can precipitate out of the solution as ferric hydroxide (Fe(OH)_3_). (Fe(OH)_3_) reduces the catalytic activity of the Fe(II) and slows down the oxidation of the MB dye. By maintaining a value of around pH 3, the Fe(II) remains in the solution, and the reaction proceeds at a faster rate. As described in the literature, the oxidation reaction strongly depends on the particle size. The smaller the nanoparticles, the larger the specific surface area, which allows for a faster oxidation reaction [43,44]. Figure 12 illustrates the impact of varying pH levels (3, 7 and 11) on the decolorization of MB dyes.

(ii) Effect of Calcium Peroxide Initial Dosage

**Figure 13:**
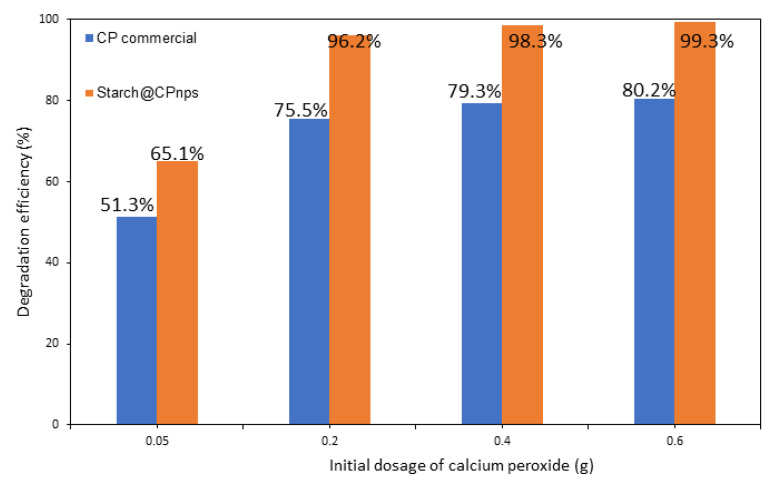
Effect of calcium peroxide initial dosage on the degradation efficiency of MB dyes; initial pH = 3, [MB]_o_ = 20 ppm, Fe(II) dosage = 0.1 g and contact time = 60 min.

The efficiency of the Fenton process, which produces hydroxyl radicals (•OH), is directly affected by the amount of CaO_2_ used as a source of •OH. Therefore, the influence of different CaO_2_ dosage (0.05, 0.2, 0.4 and 0.6 g) on the degradation of 20 ppm MB at pH 3 with 0.1 g Fe(II) at room temperature (25 ± 2 °C) was studied. The pH of the MB dye solution was kept at pH 3, as the optimal pH for Fenton oxidation has been determined in the previous section. As illustrated in Figure 13, the degradation efficiencies of MB in solution improved as the dosage of CaO_2_ increased from 0.05 g to 0.2 g. The highest degradation efficiency for both samples (CP commercial and Starch@CPnps) was observed at a sample dosage of 0.6 g. As a result, the optimal initial dosage of CaO_2_ was determined to be 0.6 g. Research conducted by Tavares et al. [45] has also shown that the formation of •OH radicals increases with an increase in the dosage of CP. The amount of reactive species, such as the concentration of iron ions (Fe(II)) and hydrogen peroxide (H_2_O_2_), also plays a role in the formation of •OH. The increase in the dosage of CP is associated with the increase in the concentration of H_2_O_2_, which acts as the oxidizing agent in the Fenton reaction. As the concentration of H_2_O_2_ increases, the number of •OH radicals generated also increases, resulting in an acceleration rate of reaction for the degradation of the MB dye.

(iii) Effect of Contact Time

**Figure 14:**
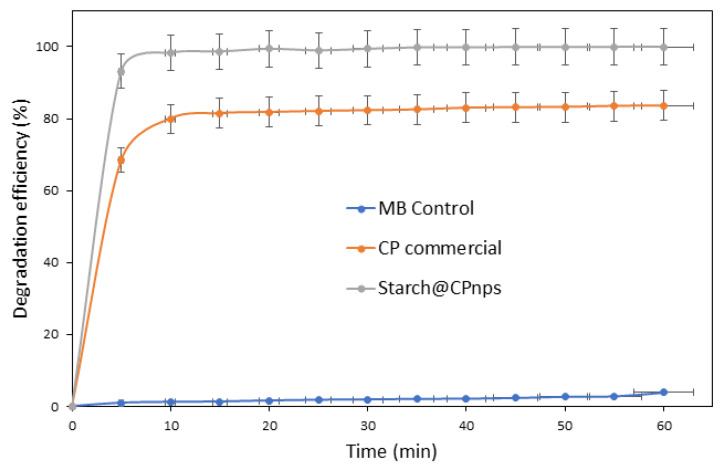
The degradation of MB dye using Starch@CPnps and CP commercial catalyzed by Fe(II), initial pH of MB dye = 3, [MB]_o_ = 20 ppm, CP dosage = 0.6 g and contact time = 60 min.

The results presented in Figure 14 demonstrate the influence of contact time on the efficiency of the degradation of the MB dye in an aqueous solution. The degradation of the dye was studied at different contact times, ranging from 10 to 60 min, using MB dye solution with an initial concentration of 20 ppm, a sample dosage of 0.6 g and an initial pH of 3 at room temperature (25 ± 2 °C). The results show that the degradation efficiency of the dye increases with time in the first 10 min and then reaches a maximum at the time of equilibrium, which is after 30 min for the 0.6 g samples. The percentage removal for the sample CP was in the range of 83% for a reaction time of 30 to 60 min and 99% for the sample Starch@CPnps. After 30 min of reaction time, both samples have no significant difference in the MB dye removal degradation efficiency. The consistency suggests that a contact time of 30 min is optimal for the degradation process, and a contact time of 60 min is the safer condition for further studies.

It is essential to study the effect of contact time, as the duration of contact between the oxidant and contaminant is a critical design parameter for the degradation process. By understanding the optimal contact time, capital and operating costs in the industry could be decreased significantly. Additionally, this information can be used to design and optimize treatment processes for the removal of other contaminants in aqueous solutions. Furthermore, the results of this study can be used to develop a more efficient and economical method for the treatment of industrial wastewater.

## 4. Kinetics Study

The data from Figure 13 were analyzed using two different kinetic models: the pseudo-first-order equation and the pseudo-second-order equation. Both models aim to describe the relationship between the degradation of the MB dye over time. The following equation (Equation (10)) represents the pseudo-first-order equation:

(10) $${\mathrm{lnC}}_{t}={\mathrm{lnC}}_{o}+k_{1}t$$

where C_t_ is the residual concentration of MB dye, C_o_ is the initial concentration of MB dye (20 ppm), and k_1_ is the rate constant, which can be calculated from the slope of the plot from Equation (10).

The pseudo-second-order equation is represented by the following Equation (11):

(1/C_t_) = (1/C_o_) + k_2_t (11)

where Ct is the residual concentration of MB dye, Co is the initial concentration of MB dye (20 ppm), and k_2_ is the rate constant, which can be calculated from the slope of the plot from Equation (11).

**Figure 15:**
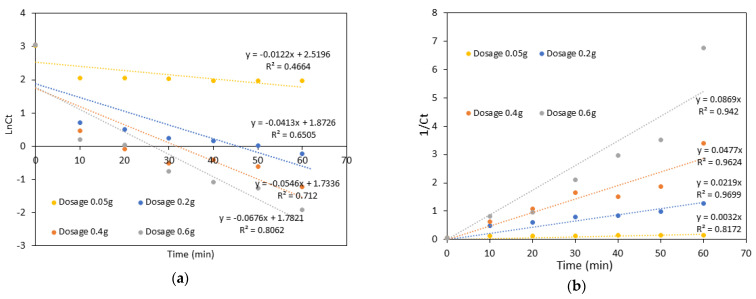
(**a**) Pseudo-first order model; (**b**) pseudo-second order model for various initial dosages of CaO_2_ ([MB]_o_ = 20 ppm, pH of MB dye = 3, Fe(II) dosage = 0.1 g and contact time = 60 min at room temperature).

The data obtained from the experiments were analysed using two different models, the pseudo-first-order and the pseudo-second-order equation. The results of the analysis are shown in Figure 15a,b, while the calculated rate constants k_1_ and k_2_ and their corresponding correlation coefficients R^2^ are given in Table 4. The results indicate that the degradation of MB dye by Starch@CPnps using the Fenton process follows the pseudo-second-order kinetics compared to the pseudo-first-order kinetics. This is due to the higher correlation coefficient R^2^ for the pseudo-second-order model. The rate constant (k_2_) increased as the Starch@CPnps dosage increased, with the highest value of 0.0869 M^−1^min^−1^. The results indicate that the pseudo-second-order model better describes the degradation of methylene blue (MB) by Starch@CPnps via the Fenton process [46].

## 5. Proposed Degradation Pathway

As shown in Equation (2), the reaction of CaO_2_ and water results in the formation of H_2_O_2_. Next, Equation (12) shows the reaction of Fe^2+^ with H_2_O_2_, where Fe^2+^ acts as a catalyst to break down the weak bond of H_2_O_2_ [47]. This reaction results in the formation of Fe(III), hydroxyl anion (^−^OH) and hydroxyl radicals (•OH), which are strong oxidizing agents [1]. •OH has an extremely nonselective behaviour and reacts rapidly with various contaminant species [48]. The hydroxyl radicals play a crucial role in the oxidation process by breaking down the MB dye into intermediate products, carbon dioxide, water and other small molecules, as shown in Equation (13) [49].

Fe^2+^ + H_2_O_2_ → Fe^3+^ + •OH + ^−^OH (12)

•OH + MB → Intermediate products + CO_2_ + H_2_O+ Cl^−^ + SO_4_^2−^ + NO^3−^ (13)

**Figure 16:**
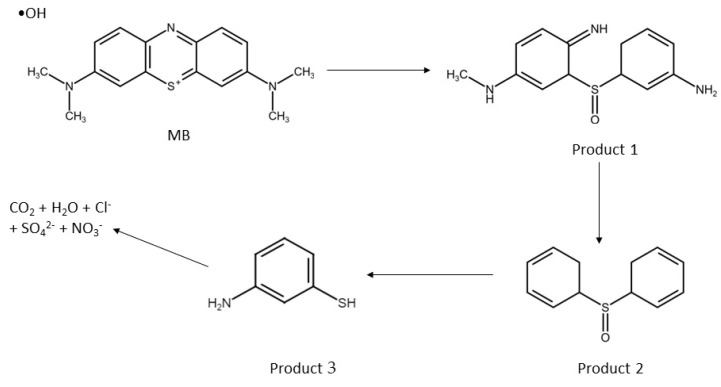
Possible degradation pathway for MB dye by Fenton reaction.

The possible degradation pathway of methylene blue (MB) dye by hydroxyl radicals (•OH) is depicted in Figure 16. The first step of the degradation process involves the formation of Product 1 by oxidation [50]. In the second step, Product 2 is formed by attacking the C—N molecular bond, which is susceptible to oxidation due to its low electronegativity [51]. In the third step, the C—S molecular bond, which has a weak bond energy, breaks and transforms into Product 3. Finally, the aromatic compound decomposes with further oxidation reactions to CO_2_, H_2_O and some inorganic ions [52].

## 6. Conclusions

In this study, the use of starch as a stabilizer to prevent the agglomeration of synthesized Starch@CPnps is explored. This study also presents the first attempt to produce calcium peroxide nanoparticles using calcium oxide as a precursor. The synthesized Starch@CPnps were characterized using various characterization techniques, such as FTIR, XRD, SEM, TGA, BET and DLS. The results showed that compared to CP commercial, the synthesized Starch@CPnps had a smaller average hydrodynamic size (47 ± 2 nm), larger surface area (35.60 m^2^/g), larger pore size (92.50 nm) and pore volume (1.65 cm^3^/g). Furthermore, the presence of starch as a stabilizer helped to maintain good aggregation stability, as evidenced by the monodisperse peak and lower PDI value (0.325) of Starch@CPnps. The degradation rate of MB dye by Starch@CPnps in an aqueous solution was also better described by the pseudo-second-order model. This study highlights the potential of using starch as a stabilizer to control the size of nanoparticles and Starch@CPnps as a promising oxidant for wastewater remediation. Further research is required to extend these findings to other contaminants, such as pharmaceuticals and organic pollutants, present in aqueous systems.

## Figures and Tables

**Table 1:** TGA summary of CP commercial, starch and Starch@CPnps.

Sample	Percentage of Residue	Major Thermal Events
CP commercial	79%	(i) 88 °C (loss of the adsorbed moisture and the decomposition of impurities) (iii) 391 °C (decomposition of calcium peroxide)
		
Starch	10%	(i) 100 °C (loss of the adsorbed moisture and the decomposition of impurities) (ii) 256–351 °C (decomposition of polymeric chains of starch)
Starch@CPnps	74%	(i) 54–183 °C (loss of the adsorbed moisture and the decomposition of impurities) (ii) 284 °C (decomposition of polymeric chains of starch) (iii) 394 °C (decomposition of calcium peroxide)

**Table 2:** List of elemental data of Starch@CPnps.

Elemental Data	Weight (%)	Atomic (%)
C%	33.34	46.30
O%	41.12	42.83
Ca%	25.23	10.48

**Table 3:** Comparison of the physicochemical properties of CP commercial and Starch@CPnps.

	Permanganate Titration	BET Analysis			DLS Analysis	
Sample	CaO_2_ content	Surface Area (m^2^/g)	Pore Size (nm)	Pore Volume (cm^3^/g)	Mean Size (nm)	PDI
CP commercial	65.0%	1.03	30.24	1.32	200 ± 100	0.561
Starch@CPnps	72.3%	35.66	92.50	1.65	47±2	0.325

**Table 4:** Comparison of degradation rate constants for Starch@CPnps using pseudo-first-order and pseudo-second-order models for various initial dosages of CaO_2_.

Sample	Initial CaO_2_ Dosage	Pseudo-First-Order Model		Pseudo-Second-Order Model	
		K_1_ (min^−1^)	R^2^	K_2_ (M^−1^min^−1^)	R^2^
Starch@CPnps	0.05 g	−0.0122	0.4464	0.0032	0.8172
	0.2 g	−0.0413	0.6505	0.0219	0.9699
	0.4 g	−0.0546	0.7120	0.0477	0.9624
	0.6 g	−0.0676	0.8062	0.0869	0.9420
Average R^2^			0.6538		0.9229

## Data Availability

The data presented in this study are available on request from the corresponding author.
